# AI‑driven photonic noses: from conventional sensors to cloud‑to-edge intelligent microsystems

**DOI:** 10.1038/s41378-025-01058-3

**Published:** 2025-11-07

**Authors:** Hong Zhou, Hemin Zhang, Ruirong Zhang, Xichen Yuan, Honglong Chang

**Affiliations:** Ministry of Education Key Laboratory of Micro and Nano Systems for Aerospace, School of Mechanical Engineering, Northwestern Polytechnical University, 710072, Xi’an, China

**Keywords:** Sensors, Optical sensors

## Abstract

The photonic nose is an emerging class of optical sensing systems designed to mimic the olfactory capabilities of a human nose. Evolving from conventional chemical and gas sensors, photonic noses leverage optical phenomena to achieve high sensitivity and fast, label-free analysis of chemical volatiles. This review provides an in-depth analysis of the evolution and current state of photonic nose technologies, particularly focusing on their integration with artificial intelligence (AI) and machine learning (ML). We first discuss key optical sensing and fabrication methods, including colorimetry, refractive index sensing, spectroscopy, and integrated photonic devices. Then, the role of ML algorithms in photonic noses is highlighted, and the integration of photonic noses into cloud-to-edge computing systems is also explored, demonstrating intelligent microsystem designs capable of on-chip real-time analytics and distributed data processing. Additionally, we highlight representative application scenarios where AI-driven photonic noses show significant advantages, including environmental monitoring, early-stage medical diagnostics, and ensuring food quality and safety. A concise comparative analysis between photonic noses, electronic noses, and analytical instruments is provided. Finally, this review identifies the remaining challenges in AI-driven photonic noses and offers insights into future development pathways toward smarter, miniaturized, and more robust photonic sensing systems.

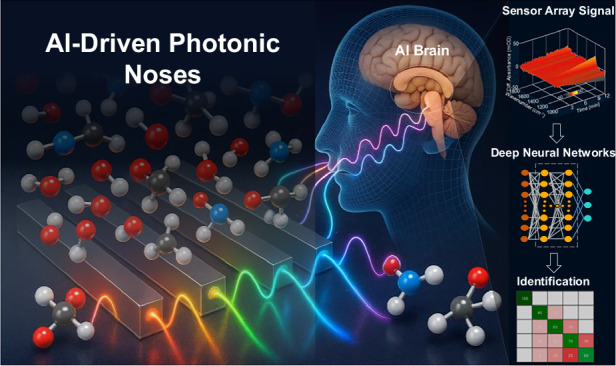

## Introduction

The ability to detect and identify complex odors and gaseous analytes is crucial in a wide range of fields, including environmental safety, healthcare, food science, and national security^1–3^. Traditional gas sensors and electronic noses (e-noses) have long been used to sense volatile compounds^4^. These e-noses typically consist of an array of chemical sensors (e.g., metal-oxide semiconductors^5^, conductive polymers^6^, quartz microbalances^7^) with partially overlapping selectivity, coupled with pattern recognition algorithms that interpret the resulting “smell” fingerprints. While successful in many applications, classical e-noses face persistent challenges^8^, including sensor drift, limited selectivity, and susceptibility to environmental factors like humidity and temperature.

Over the past decade, photonic nose technology has emerged as a powerful complement to conventional e-noses^9,10^. Rather than relying on purely electrical or chemical transducers, a photonic nose uses optical sensors and spectroscopy to detect chemicals based on their interaction with light^11^. By measuring unique absorption spectra or refractive index changes, photonic noses can achieve higher sensitivity, faster response, and improved long-term stability^12^. In essence, photonic noses mimic the biological olfactory system using photonic devices where multiple optical sensing elements act as olfactory receptors, capturing a chemical fingerprint that algorithms then interpret^13,14^, like the brain processes olfactory signals.

Early demonstrations of photonic noses were built on advances in optical gas sensing, such as non-dispersive infrared (NDIR) sensors and laser absorption spectrometers^15,16^, which target characteristic infrared absorption lines of specific gases (e.g., CO_2_ or CH_4_). Currently, the photonic noses concept has been expanded to integrate numerous optical sensors on a single platform to detect more complex mixtures and a wider variety of volatiles. For instance, integrated photonic chips can combine arrays of waveguides or microresonators, each designed or functionalized for particular analytes^17,18^. When exposed to an odor sample, these sensing elements yield an aggregate optical response (an “odor fingerprint”) that can be matched against a reference library^19^. Advances in photonic integration and microfabrication are making these systems increasingly compact and scalable, steering them toward practical intelligent microsystems that integrate both sensing and processing on a single chip.

Crucially, the rise of the photonic nose has gone hand in hand with developments in artificial intelligence (AI) and machine learning (ML). Just as the human brain interprets neural signals from olfactory receptors, AI algorithms are now harnessed to analyze the complex optical signals from photonic sensors^20–23^. Machine learning has proven indispensable for improving gas sensing selectivity and correcting sensor drift^24,25^. AI-enhanced photonic noses can resolve subtle differences in chemical mixtures, untangle overlapping spectral signals, and even quantify individual components in a multi-analyte sample^26,27^. Alongside this progression, improvements in computational resources have popularized a distributed approach to data processing. Heavy computations, such as large-scale analytics or model training, can be performed in the cloud, while lightweight AI models can operate on-edge (adjacent to the sensor) for real-time detection^28–32^. Despite these advances, AI-enabled photonic noses are still in their infancy, and merging AI with photonic nose technologies presents numerous complexities. These include issues such as sensor integration and scalability, the need for comprehensive odor datasets, and the challenge of achieving real-time, low-power operation. Significant progress has been made in addressing these challenges, though diverse technical approaches and perspectives still remain.

**Fig. 1:**
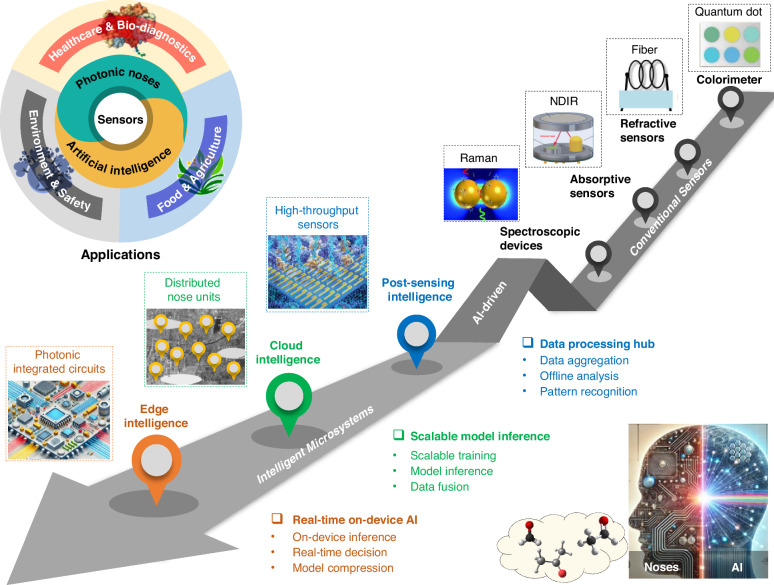
Roadmap of AI‑driven photonic noses. Early gas sensors (including colorimeters, refractive sensors, absorptive sensors, and spectroscopic sensors) paved the way for subsequent innovations. With advancements toward high-throughput sensors, distributed nodes, and on‑chip photonic integrated circuits, the post‑sensing intelligence, cloud-based processing, and edge-based intelligence have progressively been realized

This review aims to summarize key advancements in the evolution of photonic nose technology, particularly emphasizing the complexities of integrating AI into optical sensing systems (Fig. 1). First, we introduce the core technologies underpinning photonic nose systems, focusing on the fundamental principles of optical gas sensing. Next, we examine the various AI models and architectures used to process photonic nose signals, detailing the evolution from post-sensing intelligence to cloud-based and edge-based processing methods. Subsequently, we explore representative applications in environmental monitoring, medical diagnostics, and food quality assessment, illustrating how post-sensing, cloud, and edge intelligence are tailored to specific use cases. A comparative analysis is then presented to highlight the advantages of photonic noses, e-noses, and other sensor paradigms. Finally, we review the primary challenges remaining in AI-driven photonic sensing and look ahead to the future of photonic noses.

## Principle and device

### Optical sensing principle

**Fig. 2:**
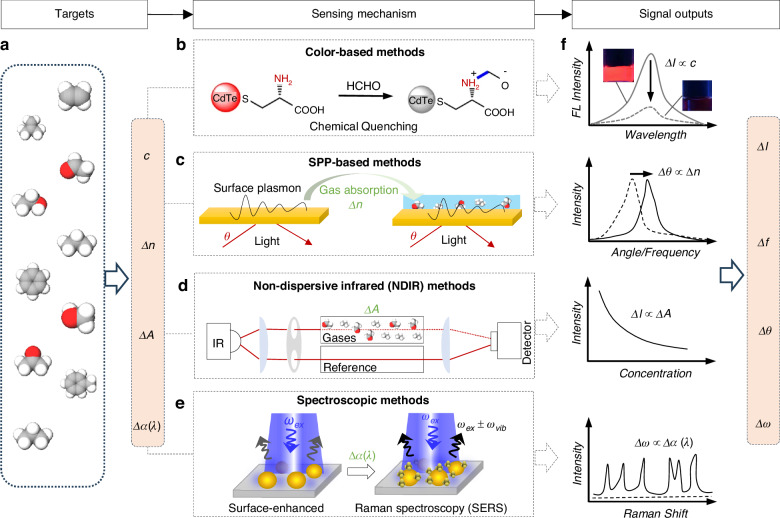
Principles of optical gas sensing. **a** Gases consist of various groups (*c*) and display distinct properties, such as refractive index (*n*), absorption coefficient (*A*) and absorption spectrum (*α*(*λ*)). **b** Colorimetric sensing mechanism: the gas groups react chemically with dye molecules, resulting in a detectable color change. **c** Surface plasmon polariton (SPP)-based methods: these techniques exploit the sensitivity of SPP to variations in refractive index (n). **d** NDIR methods: detection is achieved by measuring the absorption of infrared light by gas molecules. **e** Spectroscopic methods: for instance, surface-enhanced Raman spectroscopy (SERS) leverages Raman scattering for molecule detection. **f** Various output signal types corresponding to these mechanisms are also illustrated

Following the long-established history of optical chemical sensing, photonic nose systems detect gases and volatile analytes through direct optical interactions between light and matter. Four major optical sensing mechanisms underpin the core technologies of p-nose systems, namely sensors based on colorimetry, refractive index changes, optical absorption, and spectroscopy (Fig. 2).

Colorimetric sensors utilize measurable color changes triggered by specific chemical reactions with target gases^33,34^. Such sensors employ dyes or quantum dots (QDs) as indicators^35^, exemplified by cadmium telluride (CdTe) QDs (Fig. 2b). Certain gaseous analytes (e.g., formaldehyde) can quench their fluorescence intensity^36^, and the degree of quenching directly correlates with gas concentration, typically described by the Stern–Volmer equation.

1 $${I}_{0}/I=1+{K}_{SV}[Q]$$

where *I*_0_/*I* is intensity ratio of fluorescence before and after exposure in gases, [*Q*] represents quencher (gas) concentration, and *K*_SV_ is Stern–Volmer quenching constant. Within the linear range defined by this equation, a simple calibration curve can relate the measured intensity ratio to absolute gas concentrations. However, at higher analyte concentrations or when multiple quenching pathways coexist, deviations from linearity may occur. Therefore, modified mathematical models are required for accurate quantification under such circumstances. It should be noted that the effectiveness of colorimetric sensors relies heavily on the selection and optimization of dyes or quantum dots tailored specifically to the target analyte.

Refractive index sensors detect gases by sensing slight changes in the refractive index of the medium surrounding the optical device^37^, which is essentially the speed at which light travels. Surface plasmons (SPPs) are a typical application case^38^. An SPP is an electromagnetic wave bound to the interface between a metal (permittivity *ε*_m_) and a dielectric (*ε*_d_ = *n*^2^). Its resonance condition is set by the in-plane wave-vector:

2 $${k}_{SPP}=\frac{2\pi }{\lambda }\sqrt{\frac{{\varepsilon }_{m}{\varepsilon }_{d}}{{\varepsilon }_{m}+{\varepsilon }_{d}}}$$

Where λ is the free space wavelength. Adsorption of gas molecules onto the metal surface changes *n* (and thus *ε*_d_), leading to a measurable shift in the resonance wavelength *λ*_res_. Differentiating Eq. (2) gives the first-order wavelength shift:

3 $$\Delta \lambda ={S}_{SPP}\Delta n\,{\text{and}}\,{S}_{SPP}={\lambda }_{res}\frac{{\varepsilon }_{m}}{{({\varepsilon }_{m}+{\varepsilon }_{d})}^{3/2}}$$

Here *S*_SPP_ = d*λ*/d*n* is the bulk sensitivity, typically on the order of several hundred nm per refractive‑index unit (RIU) for noble‑metal films in the visible or near‑IR. According to Eq. 3, when gas molecules bind or aggregate on a metal surface, they cause a measurable shift in the SPP resonant wavelength or optical phase (Fig. 2c). Similar refraction-based sensing approaches include ring resonators and Mach-Zehnder interferometers^39^, whose resonance or interference conditions are also shifted by changes in the refractive index. Selectivity is commonly enhanced by functional over‑coatings engineered to adsorb target molecules preferentially, such as polymer films, metal–organic frameworks, and nanoporous layers. These coatings magnify the local refractive‑index perturbation *Δn* and thus amplify the wavelength or phase shift predicted by Eq. (3).

Absorption-based sensors detect gases by measuring the optical absorption intensity of gas molecules at specific wavelengths. A representative example is the NDIR sensor (Fig. 2d)^15,40^, widely used for detecting gases such as carbon dioxide (CO_2_) and methane (CH_4_) at room temperature and 1 atm. This sensor typically consists of an infrared light source and a detector equipped with optical filters designed specifically to isolate the strong absorption bands of target gases. The governing relationship is the Beer–Lambert law and can be expressed in logarithmic form:

4 $$A\equiv -{\text{log}}\left(\frac{I}{{I}_{0}}\right)=\varepsilon cl$$

where *I*, *I*_0_, *c*, *l*, *ε* represent incident intensity, transmitted intensity, gas concentration, optical path length, and molar absorptivity (cm^2^ mol^−1^ log^−1^). Typically, the detector measures *I*_0_/*I*, and a pre-calculated calibration curve converts this ratio to a concentration *c*. Sensitivity can be increased by expanding *l* or by employing narrowband quantum cascade or light-emitting diode (LED) sources tuned exactly to the strongest molecular line. Due to their robust structure, high stability, and ease of deployment, NDIR sensors provide rapid and reliable quantitative gas analysis without the need for chemical reaction components, making them suitable for diverse environmental conditions. Optical absorption sensors typically exhibit sub-second response times, primarily constrained by gas exchange within the measurement cell and the detector’s electronics. However, variations in temperature and pressure can influence the molar absorptivity (*ε*); thus, integration of thermistors and pressure sensors is common practice to enable automatic calibration of Eq. (4).

Spectroscopy-based sensors offer comprehensive and powerful optical detection methods^41^, providing complete absorption or scattering spectra over a broad wavelength range^42–47^. This capability enables accurate chemical fingerprinting, even in complex mixtures. Surface-enhanced Raman spectroscopy (SERS) is one notable example of such technologies (Fig. 2e, f)^48^. For purely electromagnetic enhancement, the enhancement factors G can be expressed by the ratio of SERS intensity *I*_SERS_ relative to normal Raman intensity *I*_0_:

5 $$\frac{{I}_{SERS}}{{I}_{0}}\approx {\left(\frac{|E({\bf{r}})|}{|E(0)|}\right)}^{4}$$

where *E*(**r**) is the local electric field at the molecule and *E*_0_ is the incident field. The fourth power dependence arises because both the excitation and the re-radiated Raman fields are enhanced by the local surface plasmon. Hot spot field factors |E/E_0_| (10^2^–10^3^) yield enhancement factors of 10^8^–10^12^, enabling single molecule detection under optimized conditions. Because each molecule exhibits a unique set of Raman shifts, SERS provides highly selective identification down to trace concentrations. Another important technique is photoacoustic spectroscopy (PAS)^49^, which exploits periodic thermal pulses produced by gas molecules upon absorption of modulated optical radiation (room temperature and 1 atm). These thermal pulses generate acoustic waves detectable by highly sensitive microphones. For a closed cylindrical cell in the fundamental acoustic mode, the steady state photoacoustic signal *S*_PAS_ is proportional to the absorption coefficient *α*(*λ*): ${S}_{PA}=C{P}_{0}\alpha (\lambda )$, where *P*_0_ and *C* represent incident optical power and cell constant. Because *α*(*λ*) is directly related to molecular concentration *c* via Beer–Lambert absorption, quantitative gas analysis is achieved using Eq. (5). With high-power quantum cascade lasers and low-noise micro-electromechanical system (MEMS) microphones, modern PAS systems routinely achieve sub-ppm and even ppb detection limits in palm-sized modules.

Additionally, the application of functional materials has significantly enhanced the performance of optical sensing technologies. Through precise engineering of polymers, metal-organic frameworks (MOFs)^50,51^, and nanoporous structures, selective capture and enrichment of specific analytes can be efficiently achieved^52^. Their impact is clear when viewed through sensitivity and selectivity. The affinity of the functional material for the gas increases the local analyte concentration *c*_surf_ above the gas-phase value *c*. If the partition constant is *K* = *c*_surf_/*c*, then the effective sensitivity scales as *S*_eff_ = *K‧S*_0_ (unenhanced sensitivity). MOFs exhibit *K* ≫ 1 for their target gas, giving a proportional boost in signal. In terms of selectivity, competitive adsorption follows a Langmuir isotherm^53^, that is, $\theta =\frac{{k}_{ep}p}{1+{k}_{ep}p}$, where *k*_ep_ and *p* are the equilibrium constant of gas and concentration of the gas, respectively. A functional material is tailored to increase the KEP so that the fraction of adsorbent surface covered by the target gas increases. For instance, when polyhexamethylene biguanide (PHMB) is coated on a silicon-based optical resonator, both the sensitivity and selectivity of the sensor are improved.

### Fabrication of optical sensors

The fabrication of optical sensors includes bottom-up (colloidal synthesis, self-assembly of nanoparticles, etc.)^54–60^ and top-down (lithography-based patterning, etching processes, etc.)^61–63^ nanofabrication. Next, we introduce the fabrication of representative optical sensors in detail.

**Fig. 3:**
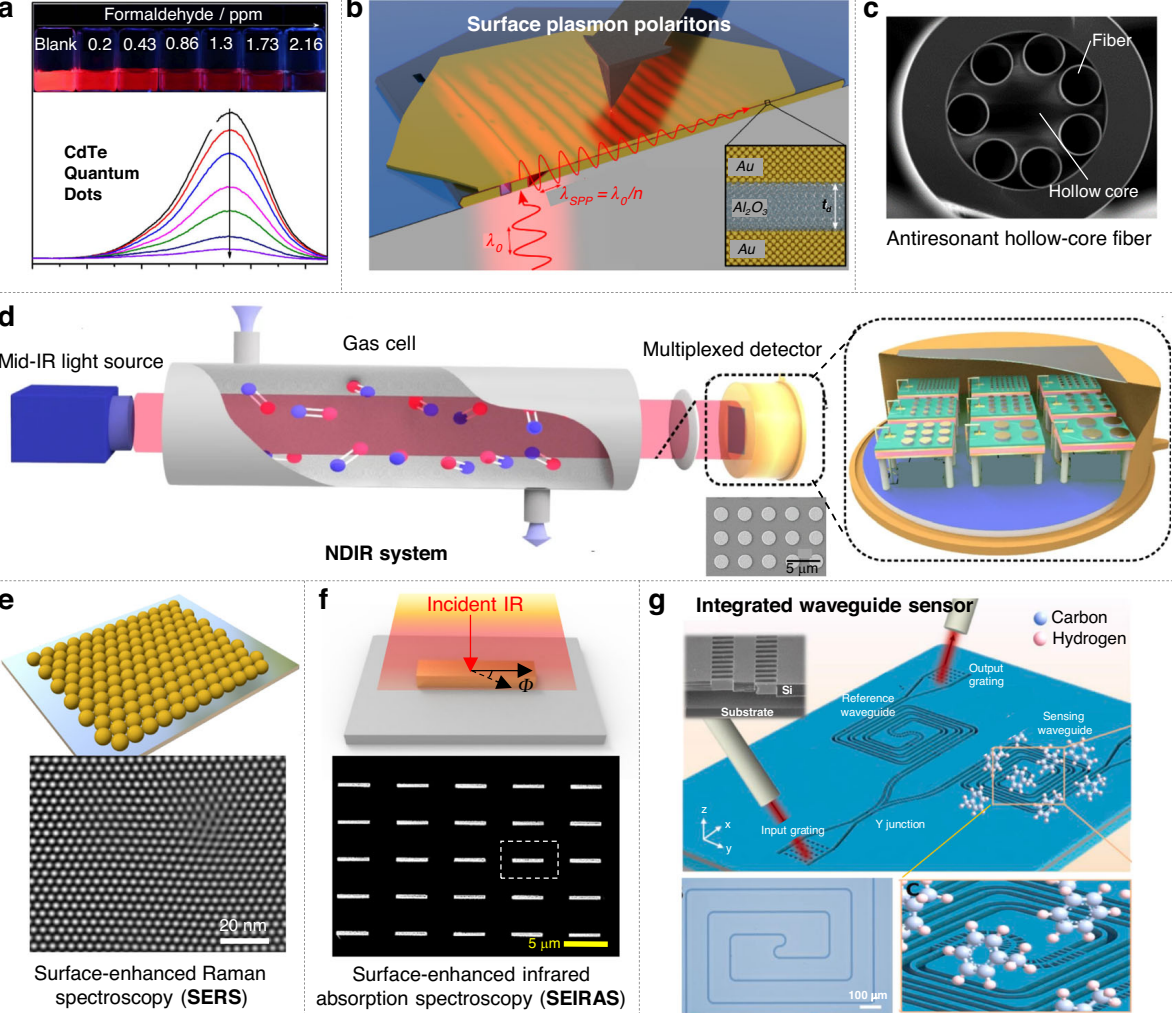
Optical gas sensors. **a** CdTe quantum dot-based gas sensor. Reproduced with permission from the Springer (2016)^66^. **b** SPP-based gas sensor. Reproduced with permission from the Springer (2022)^69^. **c** Fiber-based gas sensor. Reproduced with permission from the Multidisciplinary Digital Publishing Institute (2020)^73^. **d** NDIR gas sensor. Reproduced with permission from the Springer (2020)^40^. **e** SERS-based gas sensor. Reproduced with permission from the Springer (2024)^95^. **f** Surface enhanced infrared spectroscopy (SEIRAS)-based gas sensor. Reproduced with permission from the Springer (2023)^21^. **g** Integrated waveguide-based gas sensor. Reproduced with permission from the De Gruyter Brill (2021)^125^

Quantum-dot-based gas sensors employ nanoscale semiconductor or carbon dots as their sensing layer. Typically, quantum dots (QDs) are synthesized via colloidal chemical methods^64,65^, using materials such as metal chalcogenides (e.g., PbS, CdSe) or carbon/graphene quantum dots (Fig. 3a)^66^. After synthesis, various deposition techniques are utilized to fabricate the sensors. For instance, QD inks can be deposited onto interdigitated electrodes through drop-casting or spin-coating^67^, typically followed by mild annealing to remove residual solvents. QDs may also be incorporated into matrix materials (such as polymer blends, metal-oxide composites, and MOFs) to form robust sensing films^68^. Compared to traditional semiconductor processes, this solution-based fabrication method is relatively simple and compatible with low-temperature processing.

Early surface plasmon polariton (SPP)-based gas sensors commonly employed a configuration involving a thin metallic film deposited on a prism via physical vapor deposition, which supports collective electron oscillations at the metal–dielectric interface (Fig. 3b)^69^. Higher sensitivity SPP-based gas sensors leverage nanostructured surfaces to enhance surface plasmon resonance (SPR). These nanostructures include metal hole arrays and nanograting structures fabricated via lithography^70^, or nanoporous gold films obtained through chemical dealloying methods^71^, all of which enhance gas adsorption and plasmon propagation. Furthermore, metal nanoparticles or nanodisks, deposited via self-assembly, nanoimprinting^72^, or electron-beam lithography, are meticulously designed in size and shape to tune their resonance wavelength, significantly enhancing sensitivity towards specific gases. Overall, fabrication approaches for SPP gas sensors range from top-down lithography for creating periodic nanoarrays to bottom-up colloidal nanoparticle assembly and templated metallic growth.

Optical fiber gas sensors are based on standard fibers that undergo specialized processing for gas detection. A common approach is to expose the fiber to gases, enabling detection. For instance, when silicon-based anti-resonant hollow-core fibers are exposed to a methane and carbon dioxide mixture, their resonance wavelength shifts (Fig. 3c)^73^. To enhance the interaction between light and the target gas, several strategies are employed. First, from a materials perspective, a thin gas-sensitive layer (such as a polymer film or nanoporous material) can be coated on the fiber surface to increase gas adsorption^74^. At the device level, fabricating a tapered fiber section is an effective method^75^. By heating and stretching the fiber to form a reduced-diameter region, the evanescent field can interact directly with the surrounding gas or nanomaterials deposited on the fiber surface. Additionally, microstructured fibers, such as photonic crystal fibers with air holes^76^, can serve as gas sensors by allowing gas to permeate the air holes, thereby extending the interaction along the entire fiber length. The fabrication of these fibers involves specialized drawing techniques to form hollow cores or microcapillaries. Overall, the fabrication focuses on post-processing techniques (such as laser grating writing, etching, and side polishing) along with thin-film deposition methods like dip-coating, sputtering, and self-assembly^77–82^.

Conventional NDIR sensors typically utilize discrete components, including broadband infrared sources, gas chambers with reflective inner walls, and detectors such as thermopiles or pyroelectric sensors^83,84^. Recent advancements have focused on integrating NDIR sensors within MEMS structures^85–92^. This integration involves on-chip microfabrication of infrared sources (micro-hotplate emitters or infrared LEDs) and miniaturized detectors (thermopiles or microbolometers) coupled with optical filters^93^. For example, fully integrated MEMS-based CO₂ NDIR sensors have been demonstrated, incorporating MEMS-heated IR emitters combined with on-chip thermopile detectors arranged inside micromachined optical cavities. Such cavities are commonly etched into silicon or alternative substrates, occasionally employing hollow substrate-integrated waveguides or folded optical paths to minimize sensor size^94^. Optical filters can incorporate metasurfaces, endowing detectors with intrinsic wavelength-selective capabilities (Fig. 3d)^40^.

Surface-enhanced Raman scattering (SERS) substrates consist of engineered noble metal nanoparticles designed to amplify Raman signals via plasmonic “hotspots”. Traditional preparation methods involve electrochemical oxidation-reduction or chemical etching to create roughened metal electrodes. Alternatively, colloidal silver or gold nanoparticles (10-100 nm) can be deposited onto solid supports, forming interparticle gaps that serve as hotspots and enhance sensitivity (Fig. 3e)^95^. Advanced top-down fabrication methods, such as electron-beam or nanoimprint lithography, enable periodic structured arrays, although their effectiveness is often less pronounced compared to slightly random, cluster-based structures^96–101^. Template-assisted methods, including deposition onto porous anodized aluminum oxide or salt-crystal templates, provide a viable compromise. Commercial SERS substrates commonly consist of thin evaporated silver or gold films (5-10 nm thickness) deposited onto dielectric supports, which evolve into fractal island-like morphologies ideal for SERS enhancement^102^.

Typical surface-enhanced infrared absorption spectroscopy (SEIRAS)^103–109^ substrates are composed of thin metallic structures deposited onto infrared-transparent supports^110–117^. For instance, evaporating a gold layer onto silicon prisms for attenuated total reflection Fourier-transform infrared (ATR-FTIR) measurements creates isolated gold nanoislands resonant in the mid-infrared region, thereby significantly amplifying the absorption of surface-bound molecules. Thermal evaporation or sputtering methods, combined with thickness control and subsequent annealing, are employed to finely tune the island sizes^118^. Recent approaches have employed lithographic techniques to fabricate engineered metasurfaces and plasmonic antennas for SEIRA applications^119–122^. For example, stacked infrared nanoantennas composed of trapezoidal metallic antennas layered with phononic antennas have been fabricated using electron-beam lithography, enabling dynamic identification of overlapping molecular vibrations (Fig. 3f)^21^. Another strategy utilizes extraordinary optical transmission structures, such as periodic subwavelength hole arrays fabricated in metal films. These structures support resonant transmission modes that significantly enhance the electric field within the holes^123,124^, providing highly sensitive gas sensing through precise alignment of resonances with targeted molecular absorption bands.

Integrated waveguide gas sensors are planar photonic devices in which optical modes are confined within waveguides, interacting with gases via evanescent fields. Fabrication methods vary according to the selected platform^125–128^. Silicon photonic sensors commonly use silicon-on-insulator (SOI) substrates and rely on standard CMOS-compatible processes such as photolithography, etching, and doping to define waveguides, Michelson interferometers, ring resonators, and related components^129^. In mid-infrared applications, materials with superior optical transmission, such as silicon, germanium, or chalcogenide glasses, are preferred. For instance, a toluene vapor waveguide sensor fabricated by thermal evaporation and photolithographic patterning can detect toluene in a broad wavelength range from 6.4 to 6.8 µm (Fig. 3g)^125^. Slot waveguides require high-resolution lithography techniques to create two parallel waveguide strips separated by nanoscale air gaps, enhancing interaction through intense optical fields confined within the slot region^130^. Polymer waveguide sensors can be fabricated using molding or laser-writing methods on conventional or flexible substrates^131^, and can be integrated with dyes exhibiting optical property changes upon gas exposure.

## AI architecture for photonic nose

The raw output from a photonic nose system typically consists of high-dimensional spectral data or response vectors obtained from optical sensor arrays, containing chemical fingerprints of the sampled environment. Interpreting these fingerprints to identify and analyze complex gas mixtures represents a sophisticated pattern recognition challenge. AI and machine learning have become indispensable tools for this step^132–137^, serving a role analogous to the brain’s function in natural olfactory perception^138–156^. This section reviews the AI models and computational architectures employed in photonic nose data processing, and elaborates on the role of AI within photonic nose systems through three distinct paradigms: post-sensing intelligence, cloud intelligence, and edge intelligence.

### Post-sensing intelligence

#### Workflow of post-sensing intelligence

**Fig. 4:**
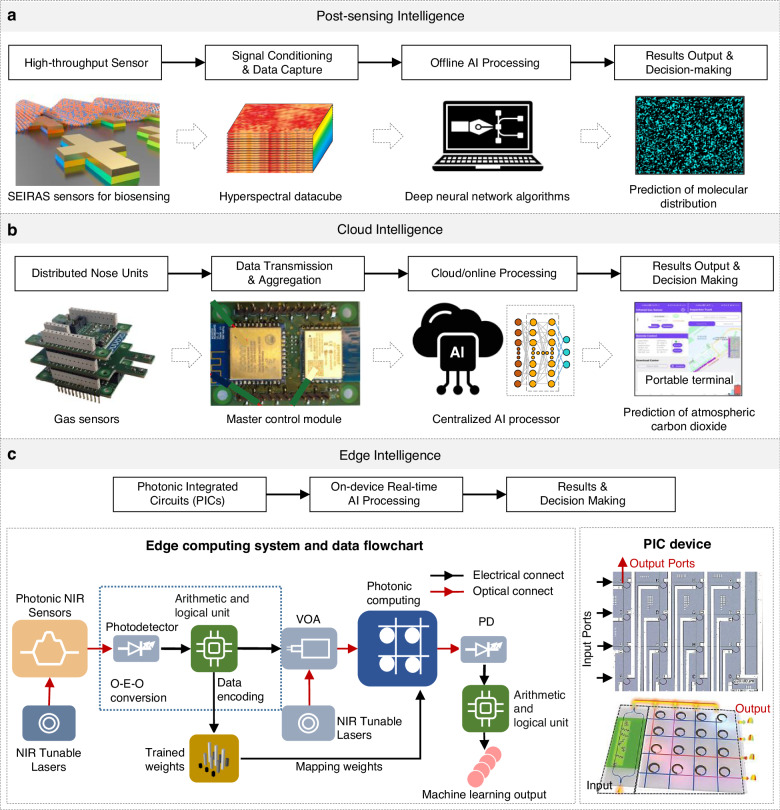
AI architecture for photonic nose. **a** Post-sensing intelligence. Reproduced with permission from the American Association for the Advancement of Science (2024)^20^. **b** Cloud intelligence. Reproduced with permission from the American Chemical Society (2025)^201^. **c** Edge intelligence. Reproduced with permission from the Wiley (2024)^212^

Post-sensing intelligence refers to the analytical process that occurs after raw signals have been captured by photonic sensors^157–160^. A typical workflow (Fig. 4a)^20^ involves three key stages^24,161–176^: (1) high-throughput measurement of gas sensor signals, (2) systematic data collection, cleaning, and annotation, and (3) application of machine learning (ML) or deep learning (DL) algorithms for data interpretation and decision-making. First, photonic nose systems generate large-scale, diverse data sets, typically containing spectral or time-resolved signals under varied analyte concentrations and environmental conditions, such as temperature and humidity variations. These datasets require preprocessing to ensure consistency, such as baseline correction, drift compensation, normalization, smoothing, and denoising, which enhances the signal-to-noise ratio and improves subsequent computational analysis. After preprocessing, the cleaned and structured data is annotated if supervised learning is applied. It is then input into the machine learning pipelines for feature extraction, analyte identification, and quantitative analysis.

#### Benefits of AI algorithms for photonic nose systems

AI methods offer significant advantages to photonic noses, addressing several critical limitations in sensors^98,168,177–184^. First, AI algorithms can automatically extract the features of the original signal. For instance, deep convolutional networks can learn hierarchical representations that capture subtle spectral motifs and slight resonance drifts^185^. Dimensionality reduction algorithms^186^, such as PCA and autoencoders, can compress sensor outputs on the order of terabytes into compact latent vectors without sacrificing the chemical specificity of gases. Second, AI algorithms can help the photonic nose suppress noise, drift, and artifacts. Optical measurements are easily affected by baseline drift and background noise derived from gas cross-sensitivity^187^. Cyclic drift compensation models can track long-term baseline drift and perform field subtraction. Anomaly detection algorithms, such as isolation forests and one-class support vector machines^188^, can flag abnormal sensor responses that indicate hardware failures. Third, AI algorithms can help the photonic nose decouple overlapping spectral features. Many volatile compounds share absorption bands or Raman peaks. Traditional univariate fitting fails once the spectra overlap. Artificial intelligence methods treat this problem as blind source separation: multi-task neural networks or physics-guided neural networks learn to decompose mixed spectra into their constituent gas components, thereby providing an accurate concentration vector for each gas^189^. Fourth, AI algorithms can help photonic noses improve selectivity and reduce detection limits. With data-driven calibration, machine learning models can leverage subtle multidimensional correlations that manual feature engineering may overlook. Ensemble learners^190^, such as gradient boosting trees and random forests, can combine weakly selective optical channels into a highly discriminative virtual receptor array, significantly enhancing class separability.

Table 1 summarizes the applications of AI and machine learning algorithms in gas sensors. Across these diverse studies, a few key conclusions emerge. First, machine learning has significantly improved sensor performance, routinely achieving over 95% classification accuracy and less than 1% concentration error, even for complex overlapping gas mixtures. Second, physics‑based approaches achieve the balance of accuracy and robustness, by combining mechanistic insights (e.g. selectivity factors, diffusion physics) with flexible algorithms (support vector machine (SVM), boosted trees, neural nets), thereby avoiding overfitting and correcting cross‑sensitivities. Third, deep architectures (convolutional neural networks (CNNs), deep belief networks (DBNs), recurrent neural networks (RNNs)) can automatically extract subtle spatiotemporal or spectral features that are hidden in noise or below conventional detection limits, enabling detection of trace gases, multi‑component speciation, and even real‑time dispersion mapping (sub‑10 ms inference). In general, post-sensing intelligence in photonic noses encompasses sophisticated machine learning algorithms that significantly elevate the capabilities of optical gas sensors.

**Table 1:** Summary of AI-enhanced gas sensors

AI algorithms	Target gases	Benefits of AI	Ref.
PSO-SVM	CO and CO_2_	Highest predictive accuracy for single/dual gas inversion; suppressed cavity-mode noise; reduced LoD to 8.2 ppmv (CO) and 13.2/4.7 ppmv (CO₂/CO)	Ref. ^182^
Machine Learning Classifier (MLC)	Common acyclic hydrocarbons	Enabled handheld FADA microspectrometer to classify hydrocarbons down to 75 ppm; single-gas LoD ~32 ppm; fast, label-free gas identification	Ref. ^26^
1D Convolutional Neural Network	H_2_O, CO_2_, O_2_, N_2_O, CO, CH_2_, NO, SO_2_, NO_2_, NH_3_	Automated feature extraction from IR spectra; speciation accuracy 82–97% on multi-component mixtures; replaces manual spectral interpretation	Ref. ^290^
Convolutional Neural Network	Benzene, Toluene, Ethylbenzene, Xylene	Precise simultaneous concentration predictions; R^2^ > 0.99 for toluene/ethylbenzene/o-xylene and >0.96 for benzene; robust against low-PEL species	Ref. ^291^
TSMC-Net (Deep Convolutional Neural Network)	Eight volatile organic compounds (VOCs)	High precision, recall, and accuracy for multigas classification from THz absorption; interpretable via class activation maps; portable THz sensor ready	Ref. ^292^
Support Vector Machine with Selectivity Factor Analysis	Mixed gases via metal-oxide sensor arrays	Established a direct proportionality between a sensor’s selectivity factor and concentration-prediction accuracy; showed that combining sensors with complementary selectivity profiles can significantly boost prediction performance	Ref. ^293^
Deep Neural Network	Hydrogen (H_2_) at concentrations below the conventional limit of detection	Extracted “hidden” sensing signals buried in noise, enhancing detection of H₂ below the traditional LOD; demonstrated universality across different sensor materials without modifying the sensors themselves	Ref. ^294^
Random Forest, ANN, k-Nearest Neighbors	Natural gas mixtures (CH_4_, two NG simulants, CH_4_ + NH_3_)	Achieved >98% identification accuracy across four gas classes; optimized model complexity to avoid overfitting; demonstrated sub-10 ms training and <0.1 ms inference on single-board hardware, enabling real-time IoT deployment	Ref. ^295^
Grey-Box SVM (physical + ML hybrid)	NO_x_ and NH_3_ in combustion exhaust	Predicted sensor outputs (NO_x_ cell current) over wide operating conditions and ammonia cross-sensitivities with high accuracy; leverages physical insight to choose only nine key features, reducing overfitting and computational load	Ref. ^296^
Boosted Regression Trees, Boosted Linear Regression, Gaussian Process	Ambient NO_2_ and O_x_ (O_3_ + NO_2_) using clustered low-cost electrochemical sensors	Combined cluster-median signals from six sensors with ML to suppress inter-sensor drift and environmental variability; ML models outperformed linear regression, producing NO_2_/O_x_ estimates comparable in RMSE to reference monitors while consuming <200 mW	Ref. ^297^
Unsupervised Neural Network with Physics-Informed Augmentation	Five gas species over 2900–3100 cm^-1^	Overcame scarce data and baseline-drift issues; achieved simultaneous identification, concentration retrieval, and pressure prediction for 31 mixtures with sub-ppb sensitivity	Ref. ^298^
Machine-Learning-Driven Wavelength Selection + Dual ANN Design	Various gas mixtures via micro-resonator arrays	Automatically selected optimal probe wavelengths based on gas absorption fingerprints; two ANNs then map wavelengths to resonator geometries, enabling compact, tunable sensor modules with precise multi-gas detection	Ref. ^28^
Stepwise Multilayer Perceptron (SMLP)	CO_2_ and CH_4_ in NDIR multipass optical measurement	Iteratively performs mixture classification then selective regression, automating feature extraction; yields 98.21% classification accuracy and normalized RMSE of 0.42%/0.45% with only 0.5 s of data and fewer training samples	Ref. ^299^
Deep Belief Network & Convolutional Neural Network	Toxic gas dispersion from point source emissions	Modeled complex dispersion fields with DBN and CNN to predict concentration contours faster and more accurately than Gaussian plume, CFD, and traditional ML models; demonstrated improved prediction accuracy for emergency response planning	Ref. ^300^
3D Convolutional Neural Network (LCNet)	O_3_ and Cl_2_ in gas mixtures via liquid-crystal film optical responses	Analyzed spatiotemporal color patterns of LC anchoring transitions to simultaneously identify O_3_ and Cl_2_ and quantify their concentrations; revealed that O_3_ is detected via transition timing and Cl_2_ via late-stage color fluctuations	Ref. ^301^
Deep-learning neural separator	CO and CH_4_	Resolves ultra-high spectral overlap via simulated training; achieves R² of 0.9996 (CH₄) and 0.9930 (CO); real-time LoD of 120.9 ppm (CH₄) and 0.5 ppm (CO); low system complexity and simultaneous detection	Ref. ^302^
VOC-Net (1D CNN)	Volatile organic compounds (VOCs)	Automates classification with >99% accuracy on simulations and 97% on noisy experimental data; provides interpretable Grad-CAM explanations	Ref. ^303^
1D CNN & deep MLP	Methane and acetylene	End-to-end concentration retrieval from direct absorption spectra; surpasses wavelength modulation spectroscopy in precision; robust to noise, laser aging, and circuit variations	Ref. ^304^
PCA, neural networks, regression	Humidity (water vapor)	Learns leaky-mode ATR reflectance dips to predict relative humidity with 0.3% accuracy using limited data; ML matches or exceeds physical-model fitting	Ref. ^305^
ML framework (multi-task + CNN)	Mixed-gas (ethylene, CO, CH_4_)	Combines tailored pre-processing, multi-task learning, and CNN architecture to significantly boost mixed-gas concentration prediction on UCI dataset compared to prior methods	Ref. ^306^
PCA, ANN, DNN, 1D CNN	Indoor VOCs: benzene, xylene, toluene, formaldehyde, ethanol	Discriminates five indoor pollutants under varying humidity and temperature; DNNs on full transients offer best performance; can reduce array to two sensors without loss of accuracy	Ref. ^307^
Temporal-based SVM with moving-window decision logic	CO, O_3_, NO_2_	Achieves 100% accuracy in both training and testing for multi-pollutant mixtures; allows user-tunable confidence thresholds to control false alarms and detection confidence	Ref. ^308^
Convolutional Neural Network	CO, NH_3_, NO_2_, CH_4_, acetone	Enables real-time gas identification with response times 1–19 s and 98% accuracy using a batch-uniform SMO sensor array; overcomes array non-uniformity	Ref. ^309^
Early-fusion multimodal AI (LSTM + CNN)	Four gas classes (via semiconductor array + thermal imagery)	Fuses gas-sensor time series (LSTM) and thermal images (CNN) to achieve 96% identification accuracy, outperforming sensor-only (82%) and vision-only (93%) models	Ref. ^310^
Recurrent Neural Network (RNN) + neuromorphic hardware synapses	NO_2_ and H_2_S	Processes sequential FET-sensor transients with 1.94% error in high-level tests; hardware RNN system runs at 0.412 mW with 0.3% overall error, ensuring low-power, reliable gas classification	Ref. ^311^

### Cloud intelligence

Post-sensing intelligence significantly improves the performance of photonic noses by locally processing raw sensor data through AI algorithms. However, sensing applications often require monitoring multiple locations simultaneously and performing long-term trend analysis. Therefore, the evolution of post-perception intelligence to cloud intelligence is crucial. With cloud-based architectures^152,191–196^, distributed sensor networks can perform extensive data integration, complex analysis, and continuous model updates.

#### Cloud architecture

A typical configuration might involve numerous photonic nose units placed in the field, and each unit is an edge device equipped with a photonic sensor, a microcontroller or processor, and wireless connectivity^197^. Data from the sensor can be transmitted directly to the cloud if the device is internet-connected. In such an architecture, there are often three layers^198^, including sensing layer, communication layer, and cloud/application layer (Fig. 4b). The sensing layer includes individual photonic nose units deployed in the field, each containing optical sensors (such as waveguides, microresonators, or spectral modules), an embedded microcontroller or processor, and wireless connectivity modules^9^. Each photonic nose locally collects spectral or refractive index data. In communication layer, data transmission from sensing units to the cloud involves communication protocols optimized for IoT applications, including Wi-Fi, LoRa, Zigbee, Bluetooth, or 5 G cellular networks^199^. The cloud/application layer performs centralized data storage, advanced analytics, and system management^200^. Sophisticated AI algorithms process large-scale aggregated sensor data, identify patterns and anomalies, and provide actionable analytics. Visualization dashboards and user interfaces present analytical outcomes to users in real-time, enabling timely decisions.

#### Distributed sensing

The development towards distributed sensing is an advantage of cloud intelligence. For instance, distributed sensing systems have been implemented using multi-sensor arrays integrated with ESP32 microcontrollers^201^. The sensors in the system capture local air-quality data and then send the measured signals to cloud platforms. The large-scale neural networks in the cloud can accurately identify various odors, quantify gas concentrations, and detect trace-level pollutants^202^. Furthermore, cloud integration enables end-users to receive real-time feedback through smartphone apps or web interfaces^203^. Distributed gas sensor networks can quickly pinpoint pollution sources by correlating data between spatially distributed devices, significantly improving the response speed and effectiveness of environmental monitoring and public health programs.

#### Cloud analytics and big data

In systems where photonic noses are widely deployed, the cloud becomes the central repository for the accumulation of massive amounts of gas sensing data. This large-scale data configuration unleashes analytical capabilities beyond local sensor processing. First, cloud-based analytics are able to identify trends, emission hotspots, and pollution sources by aggregating data from decentralized sensor nodes^204^. Second, machine learning models are continuously improved by training on large-scale data. Additionally, cloud computing enables predictive modeling by correlating real-time photonic sensor data with historical patterns and external data sets, for example, to predict air quality trends^205^. Cloud intelligence also supports continuous improvement of models through regular retraining and validation processes. Updated machine learning models are sent back to the photonic nose unit via wireless firmware updates to ensure responsiveness to emerging gases and environmental conditions.

### Edge intelligence

Although cloud intelligence provides powerful computing power and storage solutions, reliance on cloud servers brings problems, such as severe latency, higher power consumption, potential security issues, and limited functionality when network connections are unstable. Edge intelligence has emerged as an attractive alternative^191,206–211^. It enables fast and low-latency data processing near- or in- sensors, as well as fast and autonomous decision-making.

#### Edge architecture

The typical architecture of edge intelligence is integrated photonic systems, which usually consist of three key components (Fig. 4c)^212^. The first is the sensing component, which is a compact, high-performance photonic chip embedded with advanced artificial intelligence algorithms, capable of preliminary feature extraction and real-time data analysis. The second is the edge processing component, which is an efficient processor or microcontroller located near the sensor and optimized for executing lightweight machine learning models. The third is the communication component, which manages small amounts of data uploads only when necessary for further collaboration and analysis. This configuration ensures the immediate availability of actionable insights, enabling device-level autonomy.

#### Photonic integrated circuits (PICs)

PICs are a promising implementation that integrates with edge AI processors, effectively bringing computational intelligence directly into photonic chips^38^. Specifically, PICs achieve chip-level integration of photonic and electronic functions, by using silicon photonics technology and CMOS-compatible fabrication processes^213,214^. Its core components are microring resonators, Mach-Zehnder interferometers, and waveguide sensors^213,215^. By integrating these components with edge processors, AI-enhanced photonic nose systems can achieve data processing directly within the sensor. This co-design strategy allows feature extraction, pattern recognition, and classification of gaseous analytes to occur immediately upon signal acquisition, thereby eliminating communication bottlenecks and reducing energy consumption.

#### Integration of PICs with AI computing

The integration of AI computing architectures on PICs usually uses a dedicated neural network accelerator that performs complex reasoning tasks efficiently with low power consumption. Common hardware solutions include neuromorphic chips^216^, application-specific integrated circuits, and field-programmable gate arrays (FPGAs)^217^. The successful integration of AI computing architectures in photonic platforms enables real-time classification and quantitative analysis of gases with minimal latency and high accuracy. These integrated architectures are particularly beneficial for edge intelligence scenarios, where real-time, autonomous, and low-power operation are critical. In addition, advanced hybrid integration approaches are now becoming a trend. For example, mature silicon nitride (SiN) platforms are combined with lithium niobate on insulator (LNOI) or silicon-on-insulator (SOI) photonic technologies^218–220^. This hybrid integration scheme takes full advantage of the complementary advantages of each material. Silicon and silicon nitride provide powerful and mature waveguide technology and extensive electro-optical integration capabilities, while lithium niobate provides excellent electro-optical modulation speed and efficient nonlinear optical processes. This hybrid platform also helps to build compact, high-performance systems that can perform complex AI-driven tasks directly at the edge. As a result, it enables real-time environmental monitoring, medical diagnosis, and industrial process control without relying on a continuous connection to cloud resources.

## Applications of AI-driven photonic noses

### Photonic noses with post-sensing intelligence

#### Environmental monitoring

Photonic noses with post-sensing intelligence are primarily targeted at applications that require detailed data analysis and decision making after initial data acquisition and pre-processing. These applications typically involve centralized scenarios where sensor signals are processed using sophisticated machine learning algorithms to improve the accuracy and specificity of detection results.

**Fig. 5:**
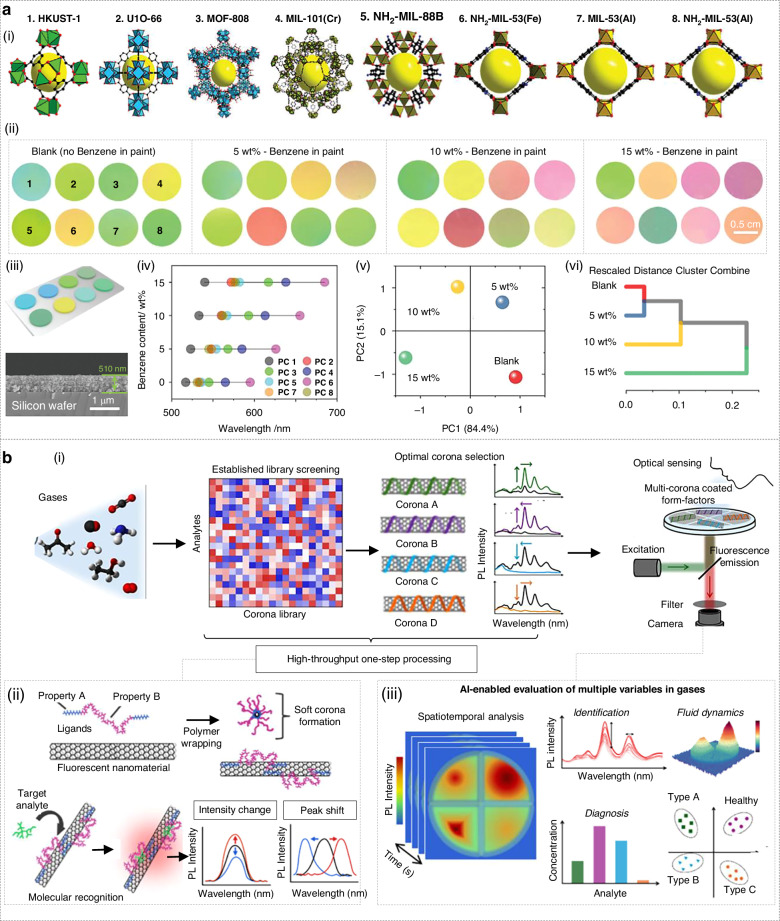
Environmental monitoring. **a** AI-enabled visual recognition of VOC gases by photonic crystals integrating multiple MOFs. Reproduced with permission from the Wiley (2024)^221^. (i) Illustration of the structure of MOFs. (ii) Photographs of the color changes of photonic nose in various benzene concentrations. (iii) Cross-sectional SEM images of the photonic nose. (iv) The photonic band gap shifts of photonic nose. (v) PCA and (vi) HCA analysis. **b** Detection of gases using AI-enhanced corona phase molecular recognition technique. Reproduced with permission from the American Chemical Society (2024)^222^. (i) The process of rapid and spatiotemporal analysis using the Corona library-driven photonic nose. (ii) Mechanism of the Corona library-driven photonic nose. (iii) AI-enabled evaluation of multiple variables in gases

A representative example is the AI-enabled visual identification of volatile organic compounds (VOCs) using a photonic nose integrated with multiple metal-organic frameworks (MOFs). Gao and co-workers demonstrated an AI-enhanced photonic nose comprising eight distinct one-dimensional photonic crystals, and each built from alternating layers of polydopamine-coated TiO_2_ nanoparticles and a different MOF (Fig. 5a, i-iii)^221^. The large surface area and tunable pore size of the MOF layer enable the selective adsorption of VOCs, which in turn changes the photonic band gap of the corresponding photonic crystal, ultimately resulting in a visible color change (photonic band gap shifts) with a sub-second response time (Fig. 5a, ii-iv). To translate these color shifts into quantitative information of gases, the diffuse reflectance spectra of all eight photonic crystals under paint-vapor exposures were captured. Then, AI algorithms, namely principal component analysis (PCA) and hierarchical cluster analysis (HCA), are then applied to classify paints according to their benzene content (0%, 5%, 10%, 15%). The results showed that PCA clearly separated four benzene content clusters in the principal component space, explaining more than 99.5% of the variance (Fig. 5a, v), while HCA separated the samples into hierarchical clusters, which were positively correlated with increasing benzene concentrations (Fig. 5a, vi).

Another photonic nose suitable for integration with AI is the corona phase molecular recognition (CoPhMoRe) technique. Rather than synthesizing multiple metal oxides (Fig. 5b, i)^222^, CoPhMoRe uses a single optical transducer, such as a near-infrared fluorescent single-walled carbon nanotube, coated with a library of synthetic polymer or biological “corona” phases (Fig. 5b, ii). These coronas form specific molecular recognition sites that change the nanotube fluorescence upon analyte binding, thereby generating a multivariate optical fingerprint for the gases. Its workflow focuses on rapidly generating hundreds of corona variants by varying polymers or biomolecules (Fig. 5, ii). Then, machine learning algorithms were used to process the complex spatiotemporal fluorescence data and correct for confounding respiratory matrix effects (Fig. 5, iii).

**Fig. 6:**
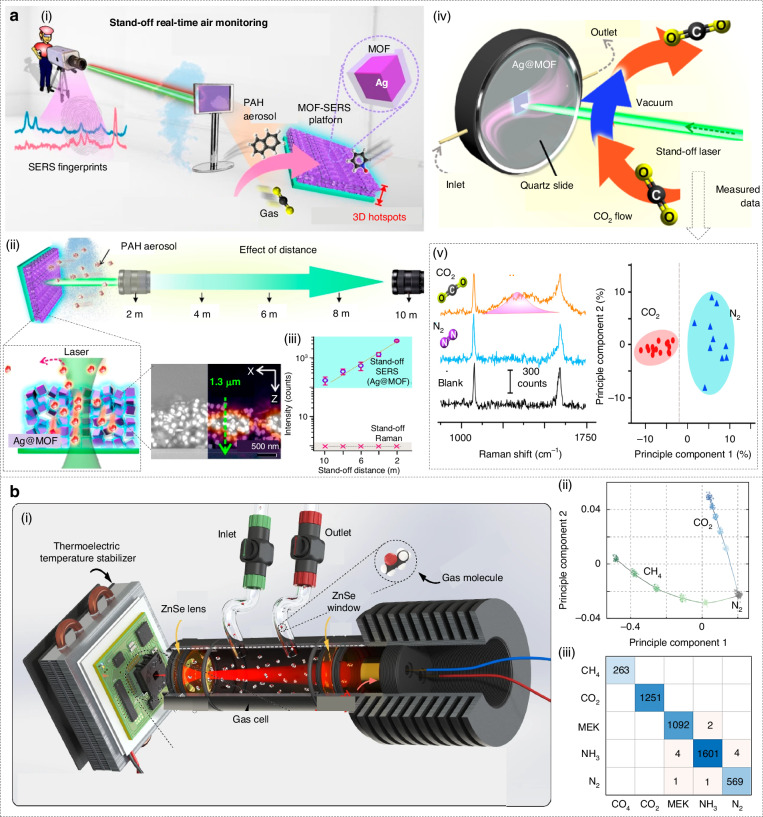
Stand-off detection technology and Smart microsystems in environmental monitoring. **a** Stand-off and real-time atmospheric monitoring using SERS-based AI photonic nose integrated with MOFs. Reproduced with permission from the American Chemical Society (2019)^223^. (i) Overview schematic display. (ii) Device characterization. (iii) Relationship between distance and signal intensity. (iv) Cycle measurement experiment. (v) Principle component analysis. **b** Smart mid-infrared microspectrometer gas sensing system. Reproduced with permission from the Springer (2024)^224^. (i) Overview schematic display. (ii) Principle component analysis. (iii) Confusion matrices of a support vector machine algorithm

Stand-off detection technology is necessary when contaminant gases are hazardous. Phan-Quang and co-workers developed a stand-off, real-time photonic nose based on 3-D metal-metal-organic-framework/Ag SERS platform (Fig. 6a, i)^223^. In the work, silver nanocubes (~121 nm) were uniformly wrapped with a sorptive ZIF-8 shell (~44 nm) and self-assembled into ∼10–15 close-packed layers, giving a ≈ 1.3-µm-thick film whose micron-scale plasmonic “hot-spot” depth matches the millimeter focal depth of the remote lens (Fig. 6a, ii). The film is interrogated with a 532 nm beam (≤55 mW) through a 200 mm lens, delivering a 1 × 1 × 3.9 mm^3^ excitation volume at distances up to 10 m (Fig. 6a, iii). During cyclic gas exposure, principal component analysis separated the CO_2_ and N_2_ classes, improving the selectivity of photonic nose despite overlapping backgrounds (Fig. 6a, iv-v).

Photonic noses integrated into small intelligent microsystems are rapidly becoming the “front-line scouts” for environmental monitoring. Their millimeter-scale size, sub-watt power consumption, and wireless connectivity mean that they can be spread across cities, factories, and forests to provide continuous high-resolution data. This development benefits from the joint development of photonics, uncooled infrared detectors, and edge AI chips. For instance, Meng and co-workers recently showcased this concept with a smart mid-infrared metasurface microspectrometer (≈1 cm³, ≈1 g) for multigas sensing (Fig. 6b)^224^. They pattern a 20-channel gold metasurface filter array directly on top of an off-the-shelf 80 × 60 micro-bolometer camera. The array tiles the 6–14 µm molecular-fingerprint window, while a 12.5 cm ZnSe gas cell and a broadband IR emitter supply the light path. A thermoelectric stage locks the detector at 28 °C, keeping baseline drift below 0.1%. The result is a consumable-free, IoT-ready microspectrometer that detects CO_2_, CH_4_, NH_3_ and methyl-ethyl-ketone down to ppm levels with >98% identification accuracy and without any moving parts or external optics (Fig. 6b, iii).

Since environmental monitoring often requires long-term use of photonic noses, robustness and drift compensation are critical. AI and machine learning techniques have become important tools to correct sensor drift, maintain calibration, and ensure the long-term reliability of photonic noses. For instance, a masked autoencoder can learn the “clean” sensor response and reconstruct the input data, isolating the drift component as reconstruction error^225^. The learned drift feature vector is concatenated with the original sensor data to provide information for the concentration estimation of the neural network. The method is verified to restore the long-term accuracy of the sensor array dataset. Besides, since there are usually differences between training data and test data, calibration of the algorithm model is also very important. Semi-supervised adversarial domain adaptation convolutional neural networks can use adversarial training to minimize the difference between labeled data and drifting unlabeled data^226^, thereby maintaining classification performance over months of operation. In addition, the long-term reliability of photonic noses can be enhanced by active query strategies. For example, the active learning framework periodically selects samples with the largest prediction uncertainty for user-guided labeling^227^, and then updates the model in real time to correct the drift.

#### Healthcare and biomedical diagnostics

Diagnosing disease by analyzing exhaled breath and body odor is a rapidly developing field, and photonic noses show great promise. Human breath contains hundreds of VOCs, some of which can serve as biomarkers for disease. For instance, acetone can be used as a diagnostic for ketoacidosis or diabetes, nitric oxide as a diagnostic for asthma, and certain aldehydes as diagnostic for lung cancer. Photonic nose systems, especially when enhanced by AI algorithms, can perform breath analysis non-invasively, which could achieve early detection of disease signatures.

**Fig. 7:**
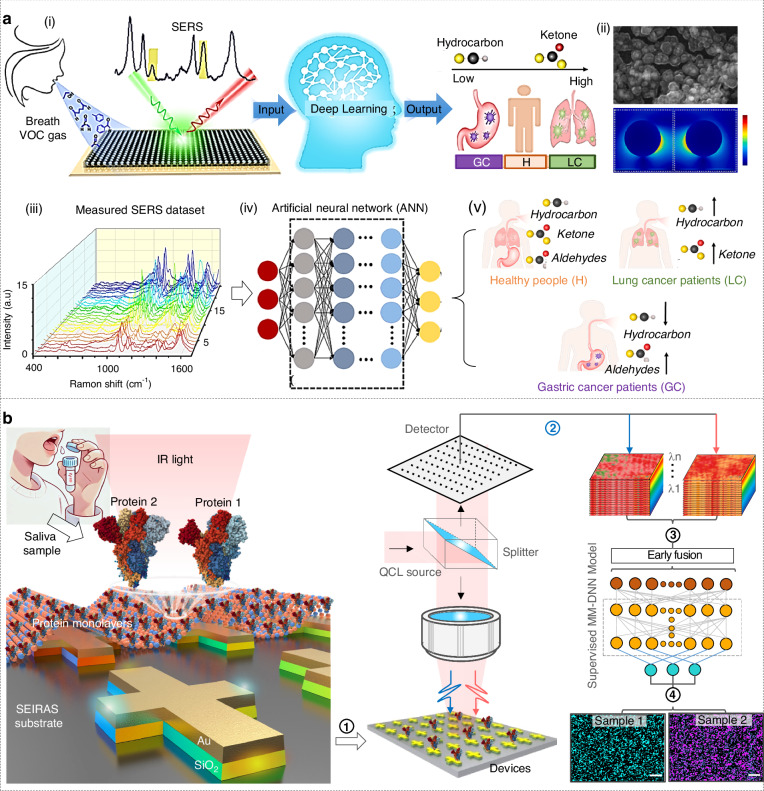
Healthcare and biomedical diagnostics. **a** SERS-based AI diagnosis of lung and gastric cancer via exhaled breath. Reproduced with permission from the Elsevier (2024)^228^. (i) Workflow. (ii) Device characterization. (iii) Measured SERS spectrum. (iv) ANN model architecture with four layers of the neural network. (v) Prediction results. **b** Workflow of AI-enhanced detection and imaging of severe acute respiratory syndrome coronavirus (SARS-CoV) spike proteins in saliva. Reproduced with permission from the Springer (2024)^20^. Workflow includes (1) sampling and immobilization, (2) spectral measurement, (3) image processing using multimodal deep neural network model, and (4) prediction

An example of biomedical diagnostics is the early diagnosis of lung cancer (LC) and gastric cancer (GC) by using photonic noses to monitor the patient’s breath gases, as demonstrated by Xie and co-works (Fig. 7, i)^228^. In this work, a plasmonic-metal–organic-framework nanoparticle (PMN) film was fabricated by self-assembling 150 nm silver core nanoparticles coated with ZIF-8 shells (20, 30, or 50 nm thick) atop a gold film (Fig. 7a, ii)^228^. To enable selective aldehyde capture, the 30 nm-shell PMN surface was functionalized with 4-aminothiophenol (PATP), which condenses with gaseous glutaraldehyde to form a C=N bond. Then, deep-learning algorithms were used for cancer classification. Specifically, a total of 1780 spectra were collected with 940 from healthy volunteers, 440 from LC patients and 400 from GC patients (Fig. 7a, iii). An artificial neural network with four hidden layers was trained (80/20 train/test split) to classify spectra into healthy (0), LC (1), or GC (2) (Fig. 7a, iv). The model achieved 97% accuracy on the training set and 89% on the test set, outperforming partial least-squares discriminant analysis. Receiver-operator characteristic curves confirmed excellent discrimination among all three classes (Fig. 7a, v).

Then, leveraging the complex biochemical signatures present in saliva, photonic platforms can non-invasively monitor viral infections with unprecedented sensitivity and speed. For instance, Zhou and co-works developed a dual plasmon–phonon nanoantenna platform on CaF_2_, consisting of asymmetric Au and SiO_2_ antennas, for the imaging of severe acute respiratory syndrome coronavirus (SARS-CoV) spike proteins in saliva (Fig. 7b)^20^. After obtaining hyperspectral datacubes using the plasmon–phonon nanoantenna platform, a multimodal deep neural network was developed, with 80% of spectra for training the classification model and 20% of spectra for testing. It achieves rapid (230,400 spectra/s) and accurate (93%) de-overlapping of two spectrally overlapping SARS-CoV spike proteins, far outperforming conventional plasmon approaches. While this case may involve analyzing liquids samples, it highlights the ability of photonic sensing combining AI technology to pick up subtle signals associated with disease.

Beyond liquid biopsies, photonic noses extend optical sensing to volatile compounds, enabling real-time monitoring of metabolic and pathological changes via breath and skin emissions. For instance, photonic nose was used for the monitoring of body odors or skin volatiles, which can change due to metabolic disorders or infections. An “electronic nose” approach has been used to sniff out wound infections or differentiate bacterial strains via their smell. A photonic nose could bring higher sensitivity and possibly identify specific molecular culprits, such as certain alcohols or amines released by bacteria. In critical care, breath photonic noses might continuously monitor sedation levels by detecting anesthetic gases or metabolites, and in sports medicine, they can track biomarkers of fatigue or hydration through breath.

#### Agriculture and food monitoring

**Fig. 8:**
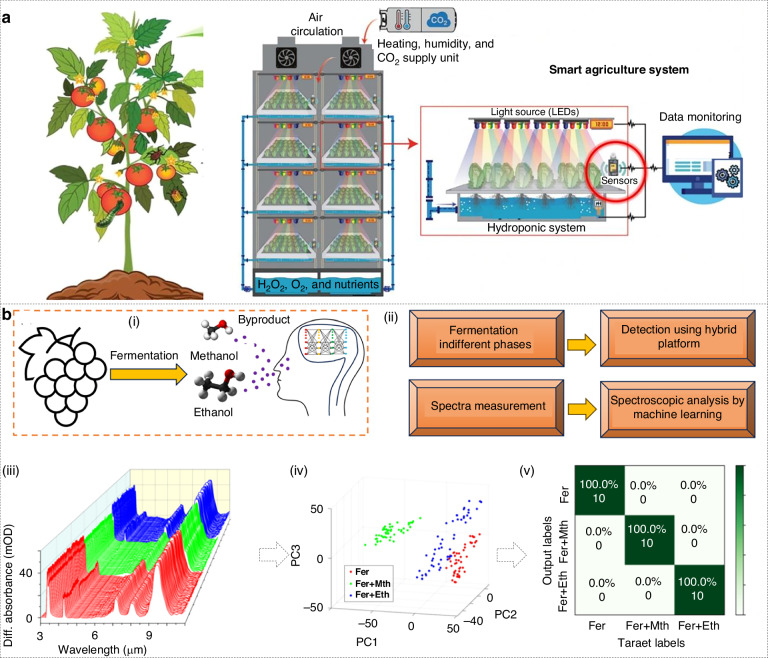
Agriculture and food quality monitoring. **a** Schematic diagram of a smart farm. Reproduced with permission from the Elsevier (2020)^229^. Smart systems consist of intelligent detection systems that can monitor temperature, humidity and gas sensors, etc.; multi-layer indoor plant growth systems; automatic control systems that can adjust the plant growth environment and soilless root environment. **b** Monitoring of wine fermentation process using proposed “photonic nose”. Reproduced with permission from the Wiley (2024)^233^. (i) Overview schematic display. (ii) Workflow. (iii) Measured spectra. (iv) PCA results of spectra data. (v) Confusion matrices

Climate change, labor shortages, and demand for pesticide-free produce are accelerating the shift from open-air farming to data-driven, fully enclosed “smart agriculture” systems. These systems rely on centimeter-scale microsensors that continuously monitor temperature, humidity, and complex mixtures of trace gases that indicate plant health. Photonic noses are emerging as the most versatile of these sensors because they reveal a complete molecular fingerprint without consumables, enabling early warning and closed-loop control (Fig. 8a)^229^. For instance, all-metamaterial detector arrays have shown sub-ppm sensitivity to ethylene gases, a key ripening hormone, promising non-invasive maturity monitoring in multilayer lettuce or strawberry racks^230^.

In addition, the smell of food and beverages is critical to product quality and consumer acceptance. Photonic noses are finding applications in food quality control. For example, the spoilage of meat is detected by sensing amines and sulfur compounds that increase as the product spoils^231^. Photonic noses can directly optically sense these gases, which improves selectivity and is not affected by high humidity and complex matrices in food storage environments^232^. In the beverage industry, photonic noses may assist in quality assurance of wine, coffee, or spirits. Each smell has a unique aroma fingerprint. Currently, photonic sensors functionalized with specific coatings can respond to flavor molecules. For instance, Xie and co-workers proposed a mid-infrared photonic nose that couples multiresonant gold nano-antennas with a porous ZIF-67 MOF, for real-time tracking of grape must fermentation (an archetypal beverage-industry process) (Fig. 8b, i)^233^. The plasmonic antenna array concentrates light into sub-wavelength hot spots, while the MOF pre-concentrates vapors within the sensing near-field, amplifying otherwise weak vibrational fingerprints. In the demonstration of non-invasive wine-fermentation monitoring, the system achieves 100% CNN classification accuracy, giving an immediate flag for toxic methanol contamination (Fig. 8b, ii-v).

Across all these applications, the common thread is that photonic noses offer a combination of broad analyte sensing and precise identification. Their optical basis gives them stability and sensitivity, while AI gives them the interpretative power needed for complex environments, that is, post-sensing intelligence.

### Applications with cloud intelligence

**Fig. 9:**
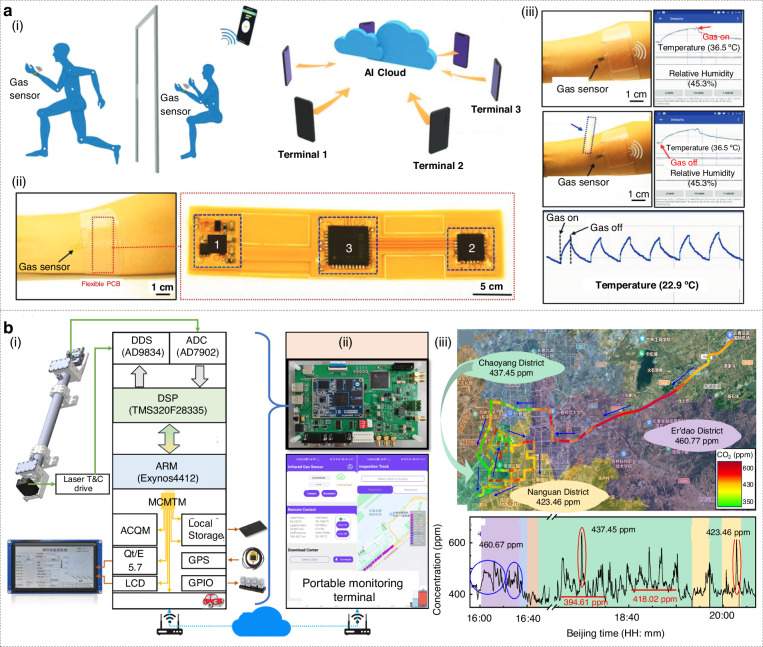
Atmospheric monitoring with cloud intelligence. **a** Cloud-based hazardous gas sensing. Reproduced with permission from the Wiley (2019)^235^. (i) Illustration of the concept of cloud data collection. (ii) Optical image of the assembly of the system. (iii) Gas response on smartphone application. **b** Near-infrared mobile cloud sensing system for atmospheric carbon dioxide monitoring. Reproduced with permission from the American Chemical Society (2025)^201^. (i) Diagram of the cloud monitoring system. (ii) Photograph of the system. (iii) The gas concentration information versus measurement time in different areas of the city

One advantage of photonic gas sensors is the high sensitivity and potential to operate in harsh conditions, as optical sensors can often be made to be resistant to corrosion and electromagnetic interference. By placing photonic noses at key points, any target gas in the atmosphere can be continuously monitored. When integrated with cloud intelligence, the data feed from each sensor becomes part of a comprehensive safety monitoring system. For instance, when a photonic sensor detected explosive gas, it could immediately send an alert to the control center via the cloud^234^. At the same time, location and severity information was sent to the worker’s smartphone. The cloud platform can also integrate inputs from multiple sensors and use models to predict the spread of hazardous gases, allowing for informed and rapid emergency response. A typical case is the development of wearable gas sensors for workers that pass measurement data to the cloud^235^. Wearable gas sensors worn on workers’ uniforms can continuously detect exposure to toxic gases and upload data in real time to a cloud dashboard. Guo and co-workers developed a thin film based on molybdenum diselenide for detecting hazardous gases and developed it with cloud-connected sensors, demonstrating how real-time cloud analysis can provide safety alerts to individuals (Fig. 9a)^235^. In an industrial setting, this means that if a worker enters an area where there is a dangerous gas leak, the system can immediately warn them and others, and perhaps even automatically activate ventilation systems.

Cloud-enabled photonic noses are also promising in environmental monitoring. This includes tracking air quality, detecting pollutants and toxic gases in the atmosphere. Environmental monitoring often requires wide-area coverage and the ability to detect multiple pollutants at low concentrations, which makes distributed sensor networks with centralized analysis capabilities particularly important. One example is the monitoring of urban air quality. Zhang and co-workers developed a compact, high-precision photonic nose based on near-infrared off-axis integrated cavity output spectroscopy, targeting the CO_2_ absorption line (Fig. 9b)^201^. A distributed-feedback laser is temperature- and current-stabilized to sweep across the absorption feature, while a 300 mm stainless-steel off-axis cavity with 99.5% mirror reflectivity yields an 891 m effective path length in just 30 cm of physical space. Signal processing relies on a dual-processor master control module comprising a DSP-based digital lock-in amplifier (DLIA) and an ARM-based multicore, multithreaded monitoring unit (MCMTM). The DLIA handles 32-bit floating-point operations to extract the absorption signal, which is then smoothed and subjected to Kalman filtering for drift compensation and noise reduction. The MCMTM runs a Linux+Qt system to manage peripherals, GPS tagging, and user interaction via a touchscreen (Fig. 9b, ii). Although no machine-learning models were deployed, the use of Kalman filtering represents an adaptive estimation algorithm critical for maintaining accuracy during mobile operation. Concentration and status data from the mobile unit are published to a central server and mirrored locally via SQLite, with a backend MySQL repository for redundancy. A companion Android app subscribes to real-time feeds, enabling remote visualization and control. Vehicle-mounted trials in Changchun city (40–80 km/h) mapped spatial CO_2_ distributions across multiple districts (Fig. 9b, iii), while a 7-day fixed-point campaign captured diurnal and weather-driven concentration dynamics.

### Applications with edge intelligence

**Fig. 10:**
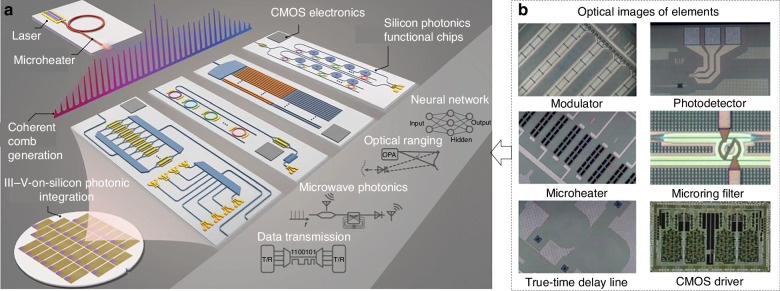
Edge intelligence based on photonic integrated circuits. **a** Conceptual image of a silicon photonic chip that enables edge intelligence, based on integrated optoelectronic systems including high-speed data transmission, microwave photonic signal processing, beam steering, and photonic computing. Leveraging III–V-on-silicon photonic integration, each chip encapsulates essential functions (laser-driven microcomb generation, passive and active optical components, and on-chip electronics) facilitating AI-driven signal processing, adaptive control, and low-latency decision-making directly at the network edge. **b** Optical images of several Si-based fundamental devices. Reproduced with permission from the Springer (2022)^237^

Silicon photonics, especially photonic integrated circuits, is a promising solution for edge computing and sensing. By fully integrating both high-speed optical data links and reconfigurable RF signal processing on a single photonic chip, this strategy embeds key intelligence functions directly at the network edge, rather than relying on distant data centers^236^. For instance, Shu and co-workers demonstrated a microcomb-driven silicon photonic system that integrates a chip-scale optical frequency comb source with standard PICs (Fig. 10)^237^. In this system, the on-chip data pre-processing and transport was achieved by modulators and detectors co-located in a compact SiPh engine (Fig. 10b). This eliminates long fiber hops to centralized servers for demultiplexing and regeneration, slashing latency and power per bit. Instead of raw analog signals traveling to a remote processor, most of the signal encoding/decoding happens right at the edge node. Besides, a tapped-delay-line microwave photonic filter is capable of dynamically reconfiguring its passband within tens of microseconds. By weighting and delaying comb lines in silicon waveguides, it performs complex RF filtering (e.g., channel selection, interference suppression) directly in the optical domain, offloading digital DSP tasks and avoiding costly electronic conversions. In terms of turnkey operation and power efficiency, the entire signal-processing chain can operate autonomously at the edge with minimal control overhead, because the AlGaAs-on-insulator microcomb source and SiPh engines require only milliwatts of on-chip pump power and no bulky stabilization electronics. By leveraging CMOS-foundry processes, additional photonic blocks, such as on-chip sensors and spectral analyzers, can be co-fabricated with microcomb sources, which is known as in-sensor computing. In essence, this approach turns a photonic link into a programmable, reconfigurable signal-processing engine, providing the hardware foundation for the edge intelligence of the photonic nose.

**Fig. 11:**
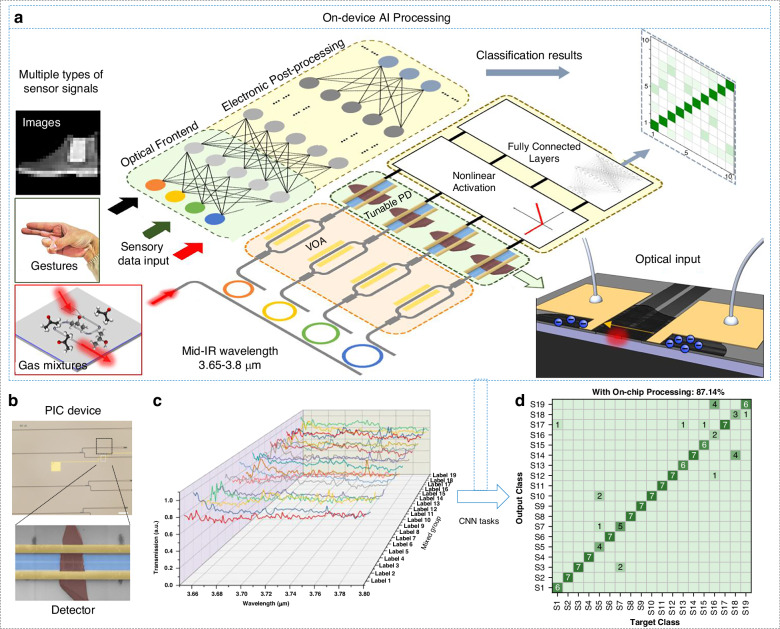
Photonic nose with in-sensor computing. **a** Schematics of the Photonic nose with an in-sensor computing unit. MIR light is coupled into the multiple waveguide channels, and each is encoded with the sensory data signal. The optical signal is then picked up by the graphene photodetector, whose weight is set by adjusting the bias voltage. The multiplied signals are then summed and sent to electrical postprocessing for classification tasks. **b** Optical images of raphene photodetector. **c** Measured Spectra of gas mixtures. **d** The confusion map of the gas mixture group classification. Reproduced with permission from the American Chemical Society (2024)^238^

The fusion of these PIC processors with optical sensors has given rise to AI-enhanced photonic noses. In post-sensor intelligence, sensor signals are transmitted to the cloud and local processors, where AI algorithms are executed to make decisions. Another state-of-the-art approach is integration of PICs with on-chip photonic sensors, performing computation within the photonic sensor hardware itself. In 2024, Liu and co-workers introduced a photonic in-sensor computing paradigm on a mid-IR silicon photonic platform (Fig. 11a)^238^. They integrated a graphene photodetector on a waveguide and exploited its bias-tunable responsivity to act as a weighted summing element (akin to a neuron) for incoming optical signals. This allowed the sensor to not only sense the spectrum but also execute neural network operations in the analog domain. Fabrication leverages CMOS-compatible processes: a 500 nm top silicon layer on 2 µm buried oxide defines the waveguides via photolithography and etching, while mechanically exfoliated graphene is transferred and patterned with Cr/Au electrodes (Fig. 11b). Characterization shows broadband, linear responsivity under ±0.4 V bias, with 16 distinct gain states (4-bit precision) and stable operation up to 5 kHz. Three proof-of-concept neural-network tasks demonstrate versatility: (1) edge-detection and classification of MNIST handwritten digits and fashion images (≈95% accuracy), (2) gesture recognition from resistive glove-sensor signals (≈72% measured accuracy), and (3) binary gas-mixture classification based on MIR absorption spectra (≈87% accuracy with 4-bit encoding) (Fig. 11c, d). This in-sensor computing approach is extremely promising because it moves the “intelligence” directly into the photonic chip, reducing the need to transmit large amounts of spectral data off-chip. Compared with cloud processing, the platform significantly reduces off-chip data transfer, lowers latency, and cuts energy consumption. Weighting and multiplication occur in the optical frontend, minimizing digital conversion overhead. The demonstrated in-sensor neuromorphic computing capability points the way to building a compact, energy-efficient, and multifunctional smart photonic nose.

Building on this proof-of-concept, a series of studies have shown how the sensing-computing continuum can be tuned to specific application needs. Liu et al. developed a photonic nose based on a millimeter-scale suspended mid-infrared spiral waveguide that captures the absorption fingerprint of isopropyl alcohol/acetone mixtures (3.65-3.80 µm)^239^. This transmits the raw spectra to cloud-hosted CNN and multilayer perceptron (MLP) models, achieving 93.6% classification accuracy for 19 mixing ratios with a concentration error of only ±2.4 vol%. On the other hand, Xiao et al. co-integrated a broadband Mach–Zehnder spectral sensor and a 4 × 4 microring resonator convolution processor to compress the entire inference pipeline onto a silicon PIC^212^. On-chip 2D convolution of the reshaped spectra was able to distinguish 45 protein/temperature classes with 97.6% accuracy, while reducing data traffic and latency by orders of magnitude. Finally, Zhuge et al. transform unavoidable photonic noise into a computational asset^240^, implementing a fully Bayesian neural network in Si/AlN. The network rivals digital precision (~98% accuracy on the MNIST dataset) and is able to intrinsically provide confidence, which is essential for safety-critical gas leak or anomaly alerts. Overall, these examples depict a range of integration strategies, from cloud-assisted spectroscopy to in-sensor convolution and Bayesian inference, highlighting how PICs can integrate sensing, processing, and trusted decision making within a single photonic platform.

Complementing these integration strategies, recent experimental benchmarks further underscore the practical viability of photonic neural-network accelerators. For example, a silicon-photonic triangular-mesh processor with in-situ backpropagation achieved over 94% accuracy on MNIST^241^. Energy-scaling analysis predicts that a 64 × 64-port implementation could be two orders of magnitude more energy-efficient than leading digital ASICs, while still delivering sustained sub-Tera- to Tera-OPS throughput, low latency, and a compact footprint. Similarly, a silicon-photonic electro-optic absorption-modulator tensor core delivered 0.108 TOPS of convolution at 2 GSa/s per channel (projected to 1.62 TOPS at full bandwidth)^242^ and achieved around 1 TOPS/W in under 1 mm² of chip area, demonstrating photonic accelerators as compact, high-throughput, and low-power solutions for integrated sensing-computing platforms. This convergence of metrics, sustained TOPS-level throughput, TOPS/W-class energy efficiency, and sub-mm^2^ integration, strongly supports the practical viability of photonic accelerators for next-generation neural-network inference.

## Comparison with other sensing paradigms

### Comparison with electronic noses

Traditional electronic noses employ sensor arrays, including metal-oxide semiconductor (MOX) sensors, conducting polymer sensors, piezoelectric sensors such as quartz crystal microbalances coated with specific sensing films, and electrochemical cells. These sensors typically indicate the presence of gases through measurable changes in electrical properties such as resistance, frequency, or current. Electronic noses have been extensively deployed in diverse applications, from environmental monitoring to medical diagnostics. However, significant challenges still exist for electronic noses, prompting increasing attention to photonic sensor technologies.

#### Selectivity

One difference between photonic noses and electronic noses is their selectivity^243^. Electronic sensors generally exhibit broad, overlapping sensitivity, resulting in ambiguous identification of specific VOCs. They rely heavily on complex pattern recognition across multiple sensors to discern individual gases. In contrast, photonic sensors inherently capitalize on different molecules’ unique spectral absorption characteristics, providing direct, intrinsic selectivity. Even in configurations utilizing broader response elements such as coated resonators, the optical signals, such as wavelength-specific shifts, provide rich datasets that machine learning algorithms can effectively decode, thereby significantly enhancing specificity compared to electronic counterparts.

#### Sensitivity

Sensitivity represents another differentiator favoring photonic noses^244^. Photonic sensors routinely achieve extremely low detection limits, frequently reaching ppm to ppb, and specialized setups even attaining parts-per-trillion (ppt) sensitivities. Optical cavity enhancement techniques and sensitive interferometric methods further elevate detection capabilities. Although some advanced electronic noses employing nanomaterial-based chemiresistors have recently improved to the ppb range, typical metal oxide (MOX) sensors predominantly operate within the ppm range. Photonic approaches offer a more robust solution by avoiding common electronic sensor limitations, such as resistance drifts and electrical noise, thus maintaining higher true sensitivity and reduced false positives.

#### Response time

photonic noses also present difference in response time^245^. Both photonic and electronic sensors can achieve rapid responses, but photonic sensors have an inherent speed advantage due to the immediate optical response upon molecular interaction. The fundamental limiting factor for both sensor types remains molecular diffusion or gas flow to the sensor surface. However, electronic sensors, particularly MOX sensors requiring heating, often exhibit slower response and recovery times, typically ranging from several seconds to tens of seconds. Photonic sensors, especially those not relying on polymer coatings with slow adsorption kinetics, consistently demonstrate sub-second response and recovery, enabling effective real-time monitoring in dynamic environments.

#### Stability and drift

Stability and drift represent chronic challenges in chemical sensing, notably in electronic noses^246^. MOX sensors often experience drift over time due to material degradation, poisoning by environmental contaminants, or sensitivity to changes in ambient humidity and temperature. Photonic sensors, built upon fundamental physical principles such as optical interference or direct absorption spectroscopy, inherently offer enhanced stability. Waveguide-based photonic sensors, constructed from inert materials, can exhibit exceptionally stable baseline performance. Although potential drift due to surface contamination or aging coatings can occur, design strategies such as protective layers and periodic self-calibration using reference gases significantly mitigate these effects. Furthermore, the reduced intrinsic drift in photonic sensors decreases the computational complexity and enhances the efficacy of machine learning algorithms used for drift compensation.

#### Cost and complexity

Cost and complexity historically favored electronic noses, owing to their relatively inexpensive sensor arrays and straightforward electronic integration. Photonic noses previously relied on costly lasers, precise optical alignment, and sophisticated optics, largely confining their use to laboratory settings^247^. This economic dynamic is evolving rapidly due to mass production of silicon photonic chips and decreasing costs of optical components such as vertical-cavity surface-emitting lasers and LEDs for specific spectral bands^248^. Although integrated photonic noses currently remain more expensive than their electronic counterparts based on commodity sensors, their higher performance, including superior sensitivity, specificity, and stability, can justify the increased investment for critical applications in medical diagnostics, security, and high-value environmental monitoring. Additionally, while photonic noses may initially require more intricate calibration procedures, their inherent stability can result in reduced long-term maintenance demands, effectively balancing the complexity considerations.

### Comparison with analytical instruments

Analytical chemistry techniques, such as gas chromatography (GC) and mass spectrometry (MS)^249–251^, are often regarded as gold standards for gas analysis, providing exceptional specificity and sensitivity. For example, GC-MS instruments can identify compounds at parts-per-trillion levels and distinguish structural isomers effectively^252^. However, these systems are typically bulky, expensive, and incapable of delivering real-time results due to analytical run times often measured in minutes. In contrast, photonic noses deliver instantaneous readings and portability, removing the need for consumables or carrier gases essential in GC-based analysis. While photonic noses cannot match the molecular separation capabilities of GC or the detailed fragmentation analysis of MS, their real-time, field-deployable functionality makes them highly practical for routine screening and continuous monitoring applications.

Another comparison involves FTIR spectroscopy, a common lab-based gas analysis method^253^. Photonic noses utilizing integrated spectroscopy effectively miniaturize FTIR-like functionality, specifically targeted at detecting predefined gases. The primary advantage of photonic noses lies in their scalability and robustness, allowing widespread distribution for continuous environmental monitoring. Deploying large numbers of compact photonic sensors for real-time monitoring of ambient conditions is feasible, contrasting starkly with the impracticality of widespread deployment of bulky benchtop FTIR instruments.

### Comparison with biological sensors

Biological sensors, such as those employing immobilized olfactory receptors or odorant-binding proteins on electrochemical or optical transducers^254^, present another intriguing comparative framework^255^. Such bioelectronic noses offer remarkable specificity, frequently at single-molecule sensitivity levels. Examples include olfactory receptors integrated onto quartz crystal microbalances or surface plasmon resonance sensors, enabling precise detection of targeted odors. However, these biological elements often face stability and longevity issues, requiring stringent conditions to preserve activity and performance.

In contrast, photonic noses employing synthetic chemistries, such as peptide-based coatings, seek to emulate biological specificity without employing delicate biological components. Biological nose systems utilizing insect antennae or cell-based sensors remain largely experimental, offering unmatched selectivity and sensitivity but limited practicality in broad applications. Photonic noses combined with artificial intelligence hold distinct advantages in consistency, robustness, and general applicability, bridging the gap between narrow-target biological specificity and broader chemical analysis needs. Table 2 qualitatively contrasts key features of photonic noses with electronic noses and GC-MS, as an illustrative comparison.

**Table 2:** Comparison of AI-enhanced photonic nose with other gas sensing technologies

Feature	Photonic Nose (AI-driven)^126,212,233,239,312^	Electronic Nose^313–315^ (MOX/polymer array)	GC-MS (lab instrument)^249–251^
Selectivity	High—uses optical spectral fingerprint or multiple specific optical channels; AI decodes mixtures.	Medium—relies on overlapping responses and AI; cross-sensitivity common.	Very High—physically separates and identifies each compound.
Sensitivity	High—sub-ppm routinely, ppb with enhancements; optical cavity methods to boost signals.	Medium—ppm typically; some nanomaterial sensors reach ppb but with noise.	Very-High–ppb to ppt for many compounds with enrichment.
Response Time	Fast—often <1 s (limited by sampling); real-time readout.	Moderate—seconds to minutes (sensor response and recovery time, esp. if heated).	Slow—minutes for chromatographic separation and analysis.
Drift /Stability	Good—optical components stable; minimal baseline drift. Periodic calibration may be needed for coatings.	Poor to Moderate—significant drift over time; frequent recalibration or baseline correction required.	Excellent per run; instrument itself needs maintenance/calibration over long term.
Power Consumption	Moderate—needs optical source and photodetectors; can be optimized for low power (edge use).	Low to Moderate—MOX sensors need heaters; others low-power; MCU usage minimal.	Very High—lab equipment with vacuum pumps, ovens, detectors, and a PC.
Size/Portability	Small—chip-scale sensors; with integrated optics can be palm-sized device or smaller.	Small—e-noses can be handheld or wearable easily.	Large—benchtop or larger, not portable in field conditions.
Data Complexity	High—rich data requiring AI interpretation.	High—multivariate data requiring AI, but typically fewer channels than photonic.	High—but processed into human-readable format by software.
Deployment Cost	Emerging—currently higher than simple gas sensors, but potential for cost reduction via mass production.	Low—many cheap sensors available; total system cost is low.	Very High—expensive instrument and skilled operators needed.
Notable Advantages	Multi-analyte detection, high specificity without reagents, fast and AI-adaptive.	Simplicity, low cost, broad response (one device can respond to many chemicals).	Definitive identification and quantification.
Notable Limitations	Still developing; might require sophisticated calibration; some designs sensitive to environmental conditions.	Prone to drift and false positives; often requires frequent recalibration; limited ability to identify specific compounds without ambiguity.	Not continuous monitoring; not suitable for on-site or real-time alerts; high maintenance.

## Conclusion and future perspectives

### Conclusion

In summary, we have comprehensively explored recent advancements in photonic nose technology, emphasizing the critical role of AI integration within optical sensing systems. Initially, we examined core technological foundations, including fundamental principles of optical gas sensing, sensor materials, and device architectures. We then detailed various AI approaches and processing architectures, tracing their evolution from initial post-sensing intelligence toward sophisticated cloud-based and edge-based processing frameworks. Through illustrative examples in environmental monitoring, medical diagnostics, and food quality assessment, we highlighted how AI-driven intelligence (spanning from centralized processing to localized edge computing) has been effectively tailored to address specific application demands. Additionally, a comparative analysis provided clarity on the unique advantages of photonic noses compared to electronic noses and traditional analytical instrumentation.

Based on these discussions, several conclusions of integrating AI with photonic nose technologies emerge clearly. Firstly, enhanced intelligence through AI enables robust pattern recognition and odor interpretation, closely mimicking biological olfactory capabilities to discriminate various odors and even accurately quantify complex gas mixtures. Secondly, AI integration allows novel functionalities, particularly through in-sensor computing and neuromorphic architectures, significantly increasing analytical capability and reducing power consumption by performing intelligent data processing directly at the sensor level. Lastly, incorporating AI into photonic noses substantially broadens potential application scenarios, facilitating seamless integration within Internet of Things (IoT) networks and cloud-to-edge computing frameworks. This integration is crucial for achieving widespread, ubiquitous sensor deployment, thereby profoundly expanding the practical reach and utility of photonic noses in diverse real-world environments.

### Challenges and future perspectives

**Fig. 12:**
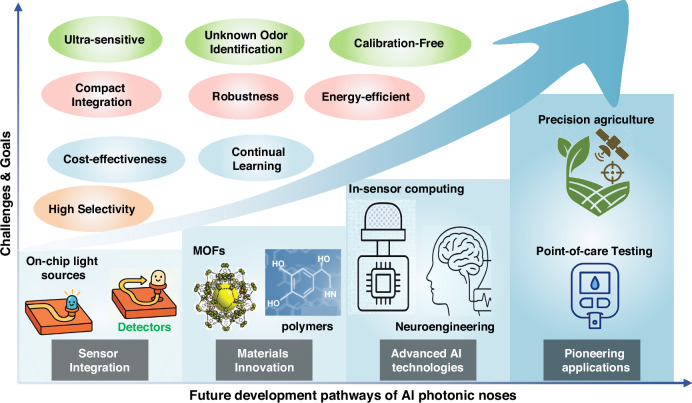
Challenges, goals, and future development pathways of AI-enhanced photonic noses

Looking forward to the development of AI photonic noses, some personal insights are proposed to promote the field to achieve sustained, high-quality progress in mechanism advancements, material innovations, technological breakthroughs, and application expansion, as illustrated in Fig. 12.

#### Sensor integration

Photonic integration allows for multiple sensing elements to be integrated onto a single chip, significantly enhancing sensor density and functionality. However, scaling these systems to large arrays remains challenging, for example, containing hundreds of sensor elements to cover a broad spectrum of analytes. Key difficulties include managing optical routing complexity, mitigating crosstalk between closely spaced sensors, and controlling insertion losses that intensify with the number of integrated components. Addressing these challenges may require the adoption of advanced strategies, such as integrating three-dimensional photonics^256^, exploring novel multiplexing techniques based on wavelength, time, or spatial dimensions^257^, in order to increase sensing capacity without degrading performance. Additionally, the development of fully integrated photonic systems, incorporating on-chip light sources and detectors, can significantly simplify packaging and improve overall reliability. Recent developments in integrating quantum cascade lasers^258–262^ and microLEDs with waveguides illustrate promising directions^101^. Furthermore, hybrid sensing approaches^263–265^, which combine photonic sensors with other devices such as chemiresistive and humidity sensors, could enhance analytical accuracy by providing complementary information that AI models can leverage. Moreover, the thermal management trade-offs inherent in integrating mid-infrared light sources with standard CMOS processes must be carefully balanced^266^. On the one hand, on-chip heaters could add complexity, footprint, and power overhead. On the other hand, inefficient heat dissipation can degrade device performance and lifespan. Equally critical is long-term device stability under variable environmental conditions^267^, since repeated thermal cycling, humidity fluctuations, and exposure to airborne contaminants can lead to drift, packaging failures, and reduced reliability over time. Future work could therefore explore advanced thermal interface materials, hermetic sealing techniques, and in-situ calibration algorithms to mitigate these effects and ensure robust field deployment.

#### Materials innovation and functionalization

The sensitivity and specificity of photonic noses, particularly those relying on refractive index sensing, depend significantly on the choice of functional materials for selectively capturing analytes. Current work on nanoporous materials, metal-organic frameworks, and advanced polymer blends aims to improve the selective affinity and reduce the detection limit^233^. Conceptually, building an expansive “material library”^206,222,268–270^ is similar to biological receptor diversity, enabling complex and unique response patterns to various chemicals. A promising concept is corona phase molecular recognition, utilizing diverse polymer coatings on carbon nanotubes exhibiting selective fluorescence responses to specific analytes^222,271^. Based on similar ideas, integrated photonic platforms can significantly expand the spatial coverage of detectable odors. In addition, advances in fabrication methods can also enhance the adaptability of devices, such as enabling reconfigurable photonic sensors to dynamically adjust resonant wavelengths or switching sensing coatings through integrated microfluidics^272^. This adaptability would significantly enhance the versatility of sensors, allowing the same device architecture to be used for a variety of applications, from environmental monitoring to security monitoring.

#### Advanced AI technologies

Advances in AI are a key area for the evolution of photonic nose technologies. The AI models that underpin the photonic nose require extensive and diverse datasets for effective training. However, obtaining comprehensive gas datasets under controlled conditions is resource-intensive, and publicly accessible standardized gas datasets remain limited. Besides, to develop robust and generalized AI models, it is essential to build an expansive library of photonic nose responses to various chemicals and complex mixtures. Additionally, transfer learning techniques are also essential, which enable AI models to be effectively adapted between different devices to compensate for subtle fabrication differences without extensive retraining^273^. Identifying unknown gases, *i.e*., samples not included in the training data, is another major AI challenge. Solutions involve employing advanced machine learning methods, such as open set classification or anomaly detection^274–279^, to classify compounds appropriately. In addition, integrating neuromorphic computing directly into the photonic nose represents an exciting frontier. For instance, combining photonic sensing with spiking neural network processing to realize a fully neuromorphic olfactory sensor^280^. It can significantly reduce power consumption and enable complex edge computing functions. Future devices may integrate analog or optical neural networks to perform preliminary calculations. The joint optimization of sensor configurations and AI models^281^, called closed-loop sensor-AI co-design, also holds great promise. It uses computational simulations to optimize sensor array designs for maximum classification accuracy and information extraction.

#### Application development

Emerging high-impact applications may significantly influence future photonic nose development. For instance, precision agriculture and food safety requirements may need more cost-effective, rugged, and user-friendly portable photonic nose solutions^282^, allowing food producers to regularly monitor crop growth conditions and storage environments. Similarly, pandemic prevention may also drive innovation in breath analysis photonic noses, enabling rapid screening of infectious biomarkers. Especially for point-of-care testing (POCT) applications^102,283,284^, it can provide real-time results with high accuracy and sensitivity, which is critical for timely diagnosis and management of diseases. In this context, microfluidic technology can be employed and integrated to enable precise sample handling and further miniaturize the overall sensing platform^285–288^. Additionally, the enforcement of environmental regulations may also drive the use of photonic noses for continuous emissions monitoring^289^, as photonic noses can provide a cost-effective alternative between manual sampling and high-cost permanent analytical instruments. Therefore, the development of photonic noses will increasingly require customized sensing ranges and sophisticated artificial intelligence analysis, designed to meet diverse and stringent specific domain requirements.
